# Family support on intensive care units during the COVID-19 pandemic: a qualitative evaluation study into experiences of relatives

**DOI:** 10.1186/s12913-021-07095-8

**Published:** 2021-10-06

**Authors:** Hanna T. Klop, Mana Nasori, Tjitske W. Klinge, Rianne Hoopman, Mirjam A. de Vos, Chantal du Perron, Lia van Zuylen, Monique Steegers, Birkitt L. ten Tusscher, Floor C. H. Abbink, Bregje D. Onwuteaka-Philipsen, H. Roeline W. Pasman

**Affiliations:** 1 Amsterdam UMC, Vrije Universiteit Amsterdam, Department of Public and Occupational Health, Amsterdam Public Health research institute (APH), De Boelelaan, 1117 Amsterdam, Netherlands; 2 Expertise Center for Palliative Care Amsterdam UMC, Amsterdam, Netherlands; 3 Department of General Practice, Amsterdam UMC, University of Amsterdam, Meibergdreef 9, Amsterdam, Netherlands; 4 Department of Human Genetics, Amsterdam UMC, University of Amsterdam, Meibergdreef 9, Amsterdam, Netherlands; 5 Department of Paediatrics, Amsterdam UMC, University of Amsterdam, Meibergdreef 9, Amsterdam, Netherlands; 6 Department of Medical Oncology, Amsterdam UMC, VU Medical Center, De Boelelaan, 1117 Amsterdam, Netherlands; 7 Department of Anaesthesiology, Amsterdam UMC, VU Medical Center, De Boelelaan, 1117 Amsterdam, Netherlands; 8 Department of Intensive Care Medicine, Amsterdam UMC, VU Medical Center, De Boelelaan, 1117 Amsterdam, Netherlands

**Keywords:** COVID-19, Critical care, Family centred care, Family support, Health care innovation, ICU, Pandemic, Relatives

## Abstract

**Background:**

During the first peak of the COVID-19 pandemic in the Netherlands, relatives of patients with COVID-19 admitted to Intensive Care Units (ICUs) were severely restricted in visiting their relatives and in communicating with treating physicians. Family communication is a core element of critical care, however, this pandemic forced medical ICU staff to arrange alternative family support for instance by Family Support Teams (FSTs), consisting of non-ICU affiliated staff who telephonically contacted relatives. This study aims to examine relatives’ experiences with FSTs on two ICUs of a Dutch university medical centre, and to evaluate its working strategies. .

**Methods:**

In a semi-structured interview study, relatives of patients with COVID-19 admitted to ICU’s, who had been supported by the FSTs, were sampled purposively. Twenty-one interviews were conducted telephonically by three researchers. All interviews were topic list guided and audio-recorded. Data was analysed thematically.

**Results:**

All participants indicated they went through a rough time. Almost all evaluated the FSTs positively. Four major themes were identified. First, three important pillars of the FSTs were providing relatives with transparency about the patients’ situation, providing attention to relatives’ well-being, and providing predictability and certainty by calling on a daily basis in a period characterised by insecurity. Second, relatives appeared to fulfil their information needs by calls of the FSTs, but also by calling the attending ICU nurse. Information provided by the FSTs was associated with details and reliability, information provided by nurses was associated with the patient’s daily care. Third, being a primary family contact was generally experienced as both valuable and as an emotional burden. Last, participants missed proper aftercare. Family support often stopped directly after the patient died or had left the ICU. Relatives expressed a need for extended support after that moment since they had strong emotions after discharge or death of the patient.

**Conclusions:**

Family support in times of the extreme COVID-19 situation is important, as relatives are restricted in communication and have a strong need for information and support. Relatives feel encouraged by structure, frequency, support and understanding by FSTs. However, remote family support should be tailored to the needs of relatives. A fixed contact person on de ICU and video calling might be good extra options for family support, also in future post COVID-19 care, but cannot replace physical visits.

## Background

In the Netherlands, the COVID-19 pandemic peaked from mid-March 2020 to the end of May 2020 for the first time [1]. Before this peak, the Intensive Care Units (ICU) in the study maintained liberal visiting hours during almost the whole day enabling relatives’ daily contact with their family member. Also, relatives received brief bedside reports on daily status by the nurses and had regularly planned contact with attending medical ICU staff. Regular strategies for ICU care delivery were undermined due to restrictions on family presence and because of outbreak management strategies combined with severe shortage of personal protective equipment (PPE). Moreover, relatives were often unable to communicate with the patient, neither by physical presence nor by digital facilities because patients were deeply sedated and receiving mechanical ventilation. In most cases, communication between relatives and treating physicians was strongly reduced because of the enormous workload of the medical ICU staff.

Family-centred care, including communication, collaboration and (bereavement) support [2], has always been a core element of ICU care, as the patients’ situation is often critical [3–5]. Precisely this family centred communication was incredibly difficult to maintain when COVID-19 in the Netherlands peaked. First published studies after the COVID-19 outbreak showed that the disease process of COVID-19 caused uncertainty among relatives, and that hospitalization of patients in complete isolation situation put an extreme burden on both the patients and their families [6, 7]. Moreover, rapid deterioration witnessed in many patients with COVID-19 admitted to the ICU [8] also caused a high burden of stress, anxiety and feelings of loneliness among relatives [9]. Therefore, support of relatives of patients with COVID-19 admitted to an ICU appeared to be extremely important.

On the ICUs of both locations of the academic hospital Amsterdam University Medical Centers, the need for alternative ways to support families in these circumstances was recognised. Therefore, on each location, a “Family Support Team” (FST) was established. Both FSTs consisted of non-ICU medical specialists from different departments and specialties. These FST members worked independently but under orders of the medical ICU staff. The FSTs aimed to provide daily medical updates about the patients situation while also guarding the family’s wellbeing and if necessary providing mental support. See Table 1 for more details of the FSTs.

**Table 1:** Overview of working strategies of FSTs

*Key elements* Daily contact by telephone with all relatives of patients admitted to this hospital’s ICU with COVID-19 from mid-March until mid-June 2020. *Goal* To provide a daily update to relatives on the patients’ situation in order to reduce pressure on ICU professionals and to provide extra support to relatives *Responsibilities of involved team members* Members of the FSTs were not part of the clinical ICU team. Team members were assigned the task of communication with the first contact person under the authority of the treating physician, except for communication regarding critical decisions such as stopping respiratory support. The ICU physician was responsible for the first contact with the relatives and introducing the FST (relatives also received written information about the FST). FST team members were granted permission the access to electronic patient files by order of the head of the ICU. **FST hospital 1** *Frequency and timing of communication* Daily telephone calls between 13:00 and 18:00 to the patients’ first contact person as mentioned in the patient file. Calls were also on weekend- and public holidays. Given information was based on the electronic patient file, in which the ICU specialist made a short note on the patients’ situation beforehand. *Team composition and working strategies* Members of the support team were a wide range of medical specialists (oncologists, anesthetists, neurologists, geriatricians). The support team was subdivided into five teams, of which each had found its own working method. Each team consisted of 4 or 5 doctors and was responsible for a certain number of families, guided by a team captain. Teams varied in composition (various medical specialist in one team or all the same medical specialists on one team) and strategy (teams who had a fixed contact person for a family or teams who had various contact persons for a family). The FST used guidelines for the conversations with relatives and a template for documenting conversations in the electronic patient file. They used a fact sheet and a list of frequently asked questions from relatives to make sure that the general information given to relatives was as consistent as possible. This information was updated regularly. **FST hospital 2** *Frequency and timing of communication* The patients’ first contact person (as mentioned in the patient file) received a daily telephone call on a specific time of their preference. This could be either in the morning, afternoon or at night. Calls were also during weekends- and public holidays. Given information was based on the electronic patient file, in which the ICU specialist made a short note on the patients’ situation beforehand about the condition of the patients and other relevant information for the family. *Team composition and working strategies* Members of the support team were all experienced pediatricians who were guided by a team captain. ICU physicians reported family contacts of newly admitted patients to the team captain and informed the family about the support team. The team captain assigned one of the pediatricians to a family contact. Support team members called families on a daily basis and made appointments about timing and amount of calls with the family contact. Support team members used the short notes of ICU physicians in the electronic patient file as background for their daily calls. The ICU physicians was available by phone for extra information if needed. Before the team started, an online meeting was held for pediatricians in which ICU physicians explained the situation on the ICU and gave information about the clinical situation of COVID-19 patients.	

This study aimed to gain a thorough insight into experiences of relatives of COVID-19 patients admitted to the ICU who had been supported by members of one of the FSTs during the first COVID-19 peak (March – June 2020) while they were not allowed to visit their relative, and to formulate suggestions for further improvement.

## Methods

### Design and participants

Two FSTs were evaluated, who both had their own operational strategy as described in Table 1.

Twenty-one semi-structured interviews were conducted with relatives who had been supported by one of the FSTs. Using purposive sampling, participants who had been called at least five times by members of the support team were included. Variation was sought with regard to: gender and age of the patient; patient deceased or not deceased; current place of stay of the non-deceased patients; type of relative (e.g. brother, child, partner), and FST. The coordinators of the FSTs made a list of all relatives who had been called, including the above mentioned characteristics. The research team selected participants from this list based on their characteristics and these relatives were first approached and informed about the interview by the coordinator of the FSTs or by the FST member who had daily contacted them, either by phone or by e-mail. If relatives agreed to be contacted by the research team, one of the researchers phoned this relative within 2 days. She gave further information and general topics of the interview, and asked if the relative was willing to participate in an interview. When the participant agreed, a date and time were set. Of 27 relatives approached, 21 actually participated. Reasons for not participating were participants being unavailable by phone (*n* = 5) or feeling too emotionally burdened by the recent experiences (*n* = 1). Characteristics of all participants are shown in Table 2. Two participants (number 18 and 21) were interviewed about two patients. Participants were mainly female (*n* = 18), partner of the patient (*n* = 10) or their child (*n* = 9). For eight participants, the relative had been deceased.

**Table 2:** Characteristics of participants interviewed

# Participant number	Relative M/F	Role	Patient M/F	Age patient	Number of calls	Patient deceased
1	F	Partner^a^	M	65–70 years	13	No
2	F	Partner^a^	M	70–75 years	33	No
3	F	Partner^a^	M	75–80 years	22	No
4	F	Child^b^	M	70–75 years	5	Yes
5	F	Partner^a^	M	70–75 years	6	Yes
6	F	Child^b^	M	70–75 years	50	No
7	M	Child^b^	F	60–65 years	8	No
8	F	Partner^a^	M	50–55 years	9	No
9	F	Child^b^	M	60–65 years	26	No
10	M	Child^b^	M	70–75 years	25	No
11	F	Sibling	F	65–70 years	25	Yes
12	F	Partner^a^	M	60–65 years	7	No
13	F	Child^b^	M	70–75 years	28	Yes
14	F	Child^b^	F	60–65 years	26	No
15	F	Sibling	F	30–35 years	15	No
16	F	Partner^a^	M	55–60 years	11	Yes
17	F	Partner^a^	M	60–65 years	31	No
18	F	Child^b^	F	N/A^c^	N/A^c^	Yes
18	F	Child^b^	M	45–50 years	18	No
19	F	Partner^a^	M	55–60 years	16	No
20	F	Partner^a^	M	65–70 years	14	Yes
21	M	Child^b^	M	70–75 years	38	No
21	M	Child^b^	F	N/A^c^	N/A^c^	Yes

^a^Ex-partners are also included in this category

^b^Children in law are also included in this category

^c^Interview was attended by another child who was not the first contact person of this relative, but who appeared to be the contact person of the other parent who had also been admitted to the ICU. As these participants were no patients at this hospital, age of the patient and number of calls are missing

### Data collection

From May 26, 2020 until September 3, 2020, data were collected among 21 participants. Fourteen interviews were conducted by one researcher (HTK), three by another researcher (MN) and four by a third researcher (TWK). All interviewers had received training in conducting qualitative interviews and had no relation to the participants. Duration of interviews varied between 16 and 56 min. Because of the COVID-19 restrictions on physical meetings, all interviews were conducted by phone or video call. Most participants preferred a telephone interview, three participants preferred an interview by video calling. All interviews were guided by a semi-structured topic list (Table 3). If relatives shared specific questions with the interviewer, for example concerning an autopsy or further contact with the physician in charge, they were referred to one of the two coordinators of the FSTs. All interviews were audio-recorded and transcribed verbatim.

**Table 3:** Topic list

Well-being of the relative and current situation	
Contact with the FST	
Structural elements (daily communication, timing, contact person) and evaluation	
Experiences with structural elements	
Themes discussed	
Being a contact person and impact^a^	
Other relatives being involved^a^	
Experiences with support of the team	
Feelings of support	
Important aspects of support	
Impact on daily life	
Expectations of FST	
Personal expectations beforehand	
Positive or negative experiences with FST	
Suggestions for future improvement	
Recommendation of this set-up for other hospitals	

^a^These themes were added to the topic list after the first three interviews.

### Analysis

All codes were inductively derived from the data, using MAXQDA 2018. The first three interviews were independently coded by two researchers (HTK and RH) and then extensively discussed by three researchers (HTK, HRWP and RH). Based on this discussion, some codes were refined and the first version of a code tree was created. In addition, the interview topic guide was slightly modified and shortened due to overlap between topics. Also, the theme *(the impact of) being a primary family contact* was added to this topic guide. Subsequently, four new interviews were coded independently and by two researchers (HTK and MN) and extensively discussed. This resulted in a further refinement of the code tree. After that, remaining data was collected and analysed inductively by three researchers (HTK, MN and TWK) until no new themes occurred from the data and the researchers therefore decided that no further data-collection was needed. Subsequently, analyses of three researchers (HTK, MN and TWK) were discussed with the supervising researchers (HRWP, MAV) in an iterative process, who critically commented on content, overlap and grouping of the themes. Finally, all themes were discussed and categorised within the research team. We followed the principles of thematic analysis [10]. The consolidated criteria guidelines for reporting qualitative studies (COREQ) were taken into account in designing and writing the article [11].

### Ethics

All participants gave oral informed consent before the interview started. Because of the COVID-19 restrictions, this spoken informed consent was given via telephone and audio recorded, which was approved by the Medical Research Ethics Committee of the Amsterdam UMC, location VUmc on May 4, 2020. This Committee determined exemption from formal review under Dutch law (registration number 2020.250). Transcripts of the interviews were anonymised to ensure participants’ privacy. Access to the data was limited to five researchers. During the interviews the interviewers paid extra attention to the well-being of the participants, given the potential emotional burden of discussing their recent experiences.

## Results

We describe the participants’ experiences regarding the FST and regarding the additional support they perceived as valuable during the first COVID-19 peak. After we searched inductively for which themes were important concerning relatives’ experiences with the FST and their working strategy, the results have been categorised in four overarching themes that emerged from the data. Themes were 1) important pillars of the FSTs, 2) combining daily calls from FST with support from the ICU ward, 3) being a primary family contact and 4) role of aftercare. In general, findings showed comparable experiences and few noticeable differences among relatives regarding the working methods of both FSTs.

Overall, relatives who were supported by the FST indicated that they went through or were still having a difficult time. Feelings of unpredictability, loneliness, stress and a rapid pace of events predominated. This is illustrated by the following quote from a participant whose husband was discharged from ICU 2 months ago.

“It was a tough time, a few weeks back. And it’s still not really... I’m still permanently worried and I haven’t yet completely recovered from the experience. It takes time to process it all. I know you can’t take life for granted anyway but what has happened recently makes you realize how vulnerable you are, not only as a human being but also as a family.” (P1, Partner, female, husband was discharged from ICU).

Furthermore, most relatives indicated that they did not know what to expect or did not expect much from the FST, and stated that the approach of the FST was beyond expectations. They highly valued that hospitals invested in this kind of family support. Moreover, most participants expressed their understanding about the lack of contact they had with the treating ICU physician due to the hectic and busy workload during the first COVID-19 peak.

“Yes. But this [video calls with the treating physician] was hardly ever possible. Well, I didn’t watch the news but of course all you ever heard all day long was that everyone in the ICU wards was rushed off their feet, and when they called I could sometimes literally hear them running or panting, you know. I mean, you’re not exactly going to say ‘Hey, make some time for me!’ It was clear they were incredibly busy.” (P16, partner, female, husband deceased).

Therefore, they supported the new approach of receiving information on the patients’ situation by the FST. Several relatives unprompted expressed that they trusted the members of the FST and their expertise. However, there were also participants who were left with unanswered questions, or had difficulties with not being able to speak to the ICU physician directly.

I: “You were called by regular specialists who were not the treating ICU physicians.”

P: “Sometimes that meant things weren’t entirely clear, because questions were left unanswered. But at least it meant the ICU doctors were spared the extra load. Some were better at finding things out than others. What you mainly want to know is why various choices were made: you want to understand that. Well, I can understand why an ICU doctor can’t take time to explain to you why they made that decision, but it would be nice if someone else can then explain it.” (P10, child, male, father was discharged from ICU)

### Important pillars of FSTs

Three elements of the FSTs appeared to be major themes, which we identified as “pillars of the support teams”. The pillars were 1) transparency about patient situation, 2) attention for relatives’ own well-being and emotions and 3) predictability and certainty; including both positive and negative experiences.

#### Transparency about the patient’s situation

Receiving a detailed medical update on the patient’s clinical status from the FST made relatives feel well informed and involved in the patients’ situation. Being able to ask questions was experienced as an essential element of the conversations and contributed to feelings of being informed and connected to the patient. Many of them appreciated detailed information and became quickly familiar with medical terms. Relatives especially appreciated the absence of time pressure when they spoke with their support team member, and his or her clarity and honesty.

“Right, the support team phoned specifically to speak to you, and when they called they had time for you. Even though they hadn’t seen my father or weren’t directly involved in his care, they could still get some information out of his medical records, or they’d just spoken to the ICU doctor on the phone or they’d already had some questions themselves, which meant they were able to tell me a lot. Sometimes they could give a bit more background information on what was going on, or explain things or say what they’d seen. So what I appreciated a lot was more the fact that they had a quiet moment, the time to give me a call.” (P9, child, female, father was discharged from ICU).

“Yes, and very honest too. Because for some things she said she simply didn’t know, she didn’t know why they were now giving him such-and-such a medicine, or why they were now putting him on a drip. She’d say, ‘I don’t know that; I’ll ask them’. And then she’d come back about it the next day. Sometimes these were urgent questions and she’d phone back that evening.” (P17, partner, female, husband was discharged from ICU).

However, some participants were disappointed that FST members could not immediately answer their questions about the patients’ situation or regarding specific characteristics of ICU care and COVID-19. Moreover, some felt frustrated that they had not been able to communicate directly to the ICU physician. They did not feel well informed because of “the second-hand” or incomplete information they had received which had raised new questions. According to a few participants, less appreciated attitudes of FST members were the variation in interpretation of medical information among FST members, as well as skills in explaining and figuring out detailed questions.

“What wasn’t so great was that I... a lot of questions didn’t get answered, purely because the doctor you have on the phone has a different specialism. You can’t blame him for that, obviously. And the ICU doctors were very busy at that time with all the cases in the hospital. One of them was very good at explaining things and finding things out if we had any questions. But that was less so with the second doctor, who was a trainee. You noticed that you didn’t feel so great afterwards then.” (P10, child, male, father was discharged from ICU).

“Well, of course [FST member’s name] can explain things as an intermediary but he’s not an ICU doctor. So he has the photos too, which he can’t show me. And he can’t explain it to me in the way a proper doctor treating the patient would because he isn’t treating the patient. So in that sense I felt I wasn’t getting an awful lot of information, but also...” (P21, children, female and male, father deceased).

#### Attention for relatives’ well-being and emotions

Feelings of powerlessness, helplessness and fear were mentioned by many participants, as well as a need for reassurance and support. These feelings arose mainly because of insecurity regarding the new virus and restrictions in visits and communication with the patient and treating physician. Participants felt that they wanted to be “held” by a healthcare provider who provided them with information, support and answers. By daily telephone contact with a member of the support team, they felt understood and taken seriously in their need for reassurance and guidance.

I: “How did you feel, being able to phone every day or getting a phone call every day?”

P: “Um, that you’re being taken seriously. Of course we’d had that unfortunate experience with our GP and now we felt hey, they are genuinely focusing on us and they’re taking it seriously. And well, basically that, kind of... sure, it’s a serious illness and new, and it wasn’t as if they always knew everything. ( …) So yes, we did get very reliable information, and I felt it was honest too, kind of ‘We’re cautiously optimistic but you need to be aware that it could take a turn for the worse again.’ But fortunately that didn’t happen. But they were very honest with us about that; they said, ‘Right we don’t know the results yet, it’s a new disease and we simply don’t know.’ So yes, I can live with answers like that. Because it says that what you don’t know, you simply don’t know.” (P12, partner, female, husband was discharged from ICU).

Relatives appreciated the personal interest. Some participants also emphasised that they appreciated the kind, human and personal attitude of their support team member. They also valued when this member openly showed his or her emotions during phone calls. Besides, relatives valued attention for their own well-being because they appreciated the feeling of being looked after and being part of care for their family member. Almost all participants indicated that they felt support from the FST among others due to the personal attention for the relative and the family as a whole.

“Yes, absolutely. I’ll be honest with you, there came a point where I thought [the physician calling] had become a family member. You know, first she would just tell me the news, about everything, but then she would also ask how I was doing, what she could do, that kind of thing. Yes... she’s a great woman, no doubt about it.” (P18, child, female, father deceased).

“P: When she phoned, she would always start by asking how I was doing and how the rest of the family was. She didn’t begin straight off with medical terms and medical information. She always asked first whether we were getting enough help and whether we needed anything. We got asked that every day.”

I: “And what was that like for you?”

P: “Well, it was nice. [...]”

I: “The very fact that people were asking this — what effect did that have on you?”

P: “Well, it was nice, to have them considering us as well rather than just the patient and the medical aspects.” (P6, child, female, father was discharged from ICU).

However, regarding attention for their well-being, other participants stated that it felt impersonal not to see the physician of the support team or that they did not feel a personal “click”, that the personal interest had felt as too much, or negatively evaluated the support team’s attention for their wellbeing, experiencing this as “playing a role”.

“At the start, I found it quite a problem, weird in the sense that he [the support team physician] was going on more about how the distance must be a problem, you living in [city] and your father being from [city], and now he’s in hospital in [city]. I thought, OK, I know all that, you don’t need to tell me. Sure, it was all well-meant but at that point I didn’t personally need it because I was more interested in right, how is he doing and how’s it going now, what are we going to do and how long is this going to go on for.”(P9, child, female, father was discharged from ICU).

#### Predictability and certainty

Relatives especially valued the structure of daily calls, and receiving an update every day. For some participants this felt as a relief, knowing that they were not left alone in uncertainty. This predictability and certainty made participants feel supported, well informed and understood in their needs.

“Um, you feel supported because you know for certain that you’ll hear every day how your loved one is doing. And that’s something you really rely on during that period. And because you’re relying on it and getting that information... well, it might sound a bit stupid, but they’re the ones who know that stuff, not me. I’m the layperson, they have the knowledge. And I have confidence in their knowledge. So you rely on that, on their knowledge. And they also know … sometimes I felt like saying, ‘Are there other things I should be asking?’ Because... do you see? Look, I don’t have that knowledge, I don’t know it all, so I... Yes, you feel supported because you’re getting information every day about someone who you can’t visit and whose life is basically hanging by a thread. And it’s really nice that they are just keeping you properly informed. That gives an awful lot of support.” (P1, partner, female, husband was discharged from ICU).

All participants recommended the support team because of certainty provided by the team, when communication with their family member and physicians often lacked. Receiving a daily update provided relatives certainty. In addition, relatives who had a permanent FST contact person were positive due to predictability and building trust. Some relatives who did not have a permanent FST contact recommended to appoint a permanent FST member for at least a couple of days, instead of different contact persons each day, while others did not perceive this as problematic. Other participants felt a lot of stress regarding the daily phone calls, due to a possibly worsening situation. A fixed moment of calls gave participants clarity, structure and peace, and met their need for guidance. However, a broad timeframe could cause stress.

“If they called at three o’clock, that was fine because then I could update my children on what the situation was. But if it was six o’clock or even later, then it was always a question of well, I still haven’t heard anything, I still haven’t heard anything. Well, then I’d be sitting on the edge of my seat all afternoon, but well... That was the only negative aspect.” (P2, partner, female, husband was discharged from ICU).

### Combining daily calls from FST with contact with the ICU ward

In addition to receiving daily calls from the FST some relatives had a second way to get fully informed on the patient’s situation through calls with the attending ICU nurse. Before COVID peaked, relatives could contact the nurse by phone for information whenever they needed this. This was also possible during the COVID crisis, and many relatives had a system of daily calling the ward nurses besides being daily informed by the FST. Contact with ward nurses was often in the form of a video call so that relatives could also see their family member. In general, the information provided by the FST was often associated with a detailed and reliable explanation, whereas the information provided by the nurses was often associated with the patient’s daily care.

Being able to video call with or via the nurse appeared to play a major role in the sense of being connected to the patient (and the nurses). Most relatives experienced the possibility to make a video call as pleasant, as they could see how the patient was doing, although the patient was mostly unconscious. It gave them a better informed picture of the patient’s situation.

“That contact was very important because you can’t visit, you can’t go there, and the video calls are a great idea, so you still get to see a bit what’s going on and how she appears. Well, perhaps most people don’t like the idea of seeing that, but perhaps some people feel a need for that and others don’t. But they ask you about that too — do you like the idea of seeing that? So it’s very good of the hospital, how they’ve arranged that.” (P15, sibling, female, sister has been discharged from ICU).

Some relatives also video called the patient if he was conscious, which helped them to feel assured and physically connected to the patient. Other relatives felt no need to video call their relative, or did not like video calling as they were afraid to see the patient lying like that and felt that video calling would be too confrontational.

“At one point I said to him [the FST physician], it feels as if he’s been dead for three weeks. I don’t hear him anymore, I don’t see him anymore. OK, you can make video calls, but I personally don’t really go for that because I think, right, you just watch someone lying asleep. That picture didn’t change much either. It wasn’t like he was suddenly lying in a different position after a week, or that he looked any different. So I found myself not really doing it [making video calls] that often.” (P9, child, female, father has been discharged from ICU).

A few of them preferred to only have contact with the patient when being conscious, another family member preferred to talk to the patient on the phone. Despite of the different experiences, all relatives found it very important to have the opportunity to either video-call or phone the patient.

### Being a primary family contact

About half of the participants indicated that they had become a primary family contact for practical reasons, e.g., skills in language, medical terms, or video-calling, while others became primary family contact because it was predetermined by previous hospitals, or because it was self-evident as a partner. Most relatives indicated being a primary family contact as heavy, intensive, and an emotional burden.

“Yes, I did find it tough. Because you... because you don’t know whether he will make it. And every time you just have to wait, every day you’re waiting to see if he’s OK. It could have been curtains at any moment because he was getting on a bit and he was diabetic. Sure, you hear all kinds of stories and see them on the TV, and you read about it in the newspaper of course. So yes, and it’s also such a horrible disease. So he’s there on a ventilator, they have to turn him over, he’s getting medicines and all sorts of things, and naturally enough they didn’t know either whether someone would make it or not. It’s not like the flu or someone who has broken his leg because you don’t know how long it will last and you always have this idea in your head that there could come a point... I knew someone who died from the coronavirus in [hospital]. They were a bit older but they died too. So yes, you’ve got that in the back of your mind. So right, every time … every time you get a call you’re kind of wow, I hope it’s good news.” (P5, partner, female, husband deceased).

Other participants expressed that they found it valuable being able to do this for their family and the patients, and even enjoyed having this kind of responsibility. The following quote illustrates the experiences of a participant, concerning being a primary family contact.

“I found it... it felt to me like something I could do for my in-laws. There’s so little you can do for one another, you know, during these Covid times – you can’t visit one another and so on. [...] Yes, I think it worked out well. And I have to say I’m pleased that it didn’t go on for months because of course it does get pretty intense after a while. But I *am* pleased that I was able to do this. It was a good thing.” (P14, child, female, mother in law has been discharged from ICU).

Almost all relatives had their own method of passing information received by the FST on to other relatives, e.g. listening together via telephone speaker, or passing on information by text message or telephone to the rest of the family.

### Role of aftercare

Some of the relatives had daily contact with the FST for several weeks and had formed a bond. This contact stopped directly after discharge or when the patient had died. Some relatives of both discharged and deceased patients expressed the termination of the daily contact as too sudden.

“My mother-in-law woke up in intensive care and she spent two days there awake I think, then she was transferred to the regular Covid ward. And then you basically go from a daily phone call about her condition to absolutely nothing. I don’t need to carry on getting a call every day but some kind of a transition would be nice because the patients are still really confused those first few days because their condition is still... they’re still very weak and their respiratory system needs to gradually get back up to scratch; I would have liked it if there’d been the same kind of phone call the next day, or two or three days later, just to bring things to a close. To have one more look at how she was doing now. Or even just from a very practical point of view: suppose we happen to have a question, where can we go to with that question? Because if we had a question and we called the ordinary nursing ward, well, that didn’t really work. Right. [...] Right, and the practical points of what now, and any questions that might come up... what’s going to happen now? And well, how... well, the fact that she’s gone from intensive care to the ordinary ward doesn’t mean that now all of a sudden... from lots of assistance to zero assistance... [silence].” (P14, child, female, mother in law has been discharged from ICU).

“Yes. Looking back, I think it would have been nice if he... well, I have to say when I came home the day he died, right, the Sunday afternoon … obviously I had an appointment with that doctor, but well, that... it was at that time that he was dying, right. And then she phoned me late that afternoon. To offer her condolences, let’s say. And she was ever so sweet about it because of course you don’t know one another at all, but I was very... I wasn’t there at all, I was a spectator, let’s say. I didn’t realize at all that this was about me [...]. And yes, in the end it would have been nice to get one more phone call, a month later for example.” (P16, partner, female, husband deceased).

Participants recommended a more gradual reduction of contact with the FST or the availability of aftercare to help relatives cope with their situation. This was relevant for both family of discharged and deceased patients, because both situations caused a major change in family contact and some of the relatives had daily contact with the FST for a rather long period of time.

## Discussion

We aimed to investigate experiences of relatives of COVID-19 patients admitted to the ICU who had received support of a family support team (FST) during the first COVID-19 peak, and to formulate suggestions for further improvement. Before the COVID-19 pandemic, both ICUs involved in this study had an open visitation policy. However, when COVID-19 for the first time peaked at March–June 2020, visitation and communication between relatives and patients was severely restricted, and family was supported by FSTs. We found that almost all relatives experienced the information and support of the FSTs as positive, humane and supportive, especially due to transparency about the patients’ situation provided by the FST, as well as attention to relatives’ well-being and providing predictability and certainty by calling on a daily basis in a period characterised by insecurity. We also found that besides receiving information from the FST, relatives independently contacted the ICU nursing staff. Combining calls of the FST and calling the ICU nursing staff appeared to be complementary and was much appreciated by relatives. The offer of video calling options was also appreciated by relatives, although not every relative wanted to use these options. Being a primary family contact was generally experienced as both valuable and as an emotional burden. Lastly we found that aftercare fell short. Studies before COVID-19 already showed that the possibility to visit patients at the ICU is important for families and it is found that an open visitation policy versus a restricted (in visiting times and number of visitors) visitation policy is associated with improved patient and family satisfaction, and reduced anxiety and depression among family members [12, 13]. Therefore, the alternative of updating and supporting relatives at distance at the time visiting patients at the ICU is not possible, is important.

We found that relatives felt supported by the daily phone calls by the FSTs. Furthermore, relatives who independently could contact the ICU nursing staff (next to the daily calls of the FST) were positive, since the ability to video call with or via the nurse gave them a sense of being connected to the patient and the nurses. However, we also found points for improvement for future similar situations:

First, the working strategies of the two studied FTSs differed (see Table 1), and families appreciated fixed or small timeframes of contact and continuity in FST contact person. These features were highly appreciated when present and gave relatives a feeling of certainty. When not present, they were experienced negatively by relatives. These results were also found in a similar study that evaluated a Family Liaison Team on an ICU in the UK [14]. Therefore it is recommended to use small timeframes and a fixed contact person as much as possible in future situations.

Second, relatives described difficulties coping with the sudden stop of daily calls after discharge or death of a patient and this often went along with complicated and intense emotions. It is already known that relatives of patients who had been admitted to the ICU may experience permanent (psychosocial) complaints, e.g., trauma or disturbed grief [15–18]. Given the unique circumstances and uncertain prognoses of COVID-19, restricted communication between physicians, patients and relatives, and separation during the COVID-19 peak, it is expected that many complaints will be complex and manifest themselves in the near or distant future [19–21]. Therefore, (prolonged) aftercare is especially important and relevant, as also plead for by Eisma et al. [22]

This study identified important elements of good aftercare that relatives missed, which are 1) receiving information after discharge or death, e.g. about recovery, or autopsy 2) assistance in processing the patients’ disease or death in isolation, e.g. by contact with medical ICU staff or by the opportunity to visit the ICU where the patient was admitted to. But also just another call from the FST after discharge of death of the patient can help families.

After the first COVID-19 peak, it was (inter) nationally evaluated that visiting policies should change (being less stringent). In the ICUs of our study, visiting the ICU was possible again as from June 2020, but still with restrictions (starting with a maximum of two persons for 1 h per day, and later on two persons for 2 h in the afternoon and two persons for 3 h in the evening). The FSTs stopped with their support when visiting was possible again. Although visiting policies are now less restricted and will hopefully return to normal we can learn valuable lesson from this period in regard of family support.

A fixed contact person gave relatives a feeling of certainty and it would be good to strive for a constant member of staff to inform and support the family. In daily practice this might however be challenging and medical social workers, spiritual caregivers or others could play an important role in supporting families. One of the ICUs in our study has therefore started with two dedicated family support workers.

Another improvement could be video calling. Video calling already happened before COVID-19, but was more structural used during the COVID-19 pandemic to replace physical contact between patient and family. Several evaluation studies about video calling with relatives of hospital patients during the COVID-19 pandemic show that relatives are overall positive about this feature, but also experienced barriers, such as technical difficulties and technological literacy (both by relatives and staff), difficult and unstructured communication, difficulty in building rapport, and lack of continuity (no consistent contact person) [23–26]. These studies also show that relatives stated that video calling cannot replace physical visits of family. Taking these barriers in consideration, video calling can be a good extra way of supporting relatives of ICU patients in the future. Based on the experiences during COVID-19, instructions and tools for video conversations with relatives, including ways to overcome the abovementioned barriers are published and can be used to implement video calling in daily practice [25, 27].

### Future research

This pandemic has shown the importance of family-centred care and suggestions for improving daily support of relatives and remote support via phone or video calling seems to be an option for extra care. However, we also found participants in our study who had rather negative experiences with the FSTs. This study therefore also raises the question whether FSTs and video calling are appropriate for all relatives and their needs. More research is needed on how to tailor this care, for instance how to determine the appropriateness of when to offer remote support via phone or video calling and to whom. Also barriers with video calling found in other studies should be studied further.

Furthermore, it could be studied whether introducing a fixed contact person at the ICU for times when relatives can visit the ICU again. Is this feasible, does this improve family satisfaction and does it give a higher feeling of certainty?

### Strengths and limitations

This study provides rich data on relatives’ experiences with the FSTs during the first peak of COVID-19 in the Netherlands and provides recommendations for further improvement of this novel type of family support. One strength of this study is the quick and thorough evaluation of alternative family support, directly during the first peak of COVID-19 in the Netherlands. This evaluation provides useful insights into best practices and also into further needs for improvement. Another strength of this study is the heterogeneity in themes and recommendations, although participants were supported by two different and independently working FSTs. Differences between working strategies of both teams show that relatives appreciate some strategies over others, such as a fixed time frame. It can be considered both as a strength and as a limitation that all 21 interviews were conducted by three researchers, which all had a personal style and background in interviewing. The teamwork and coding process ensured that the researchers learned from each other’s interview style. However, differences between questioning the topics, and therefore emphasis in the data, cannot be exempted. Another limitation could be found in the perspective of this study, which focused on relatives’ experiences. Little is known from the perspective of members of the support teams, medical ICU staff and other relatives who were not the primary contact person. It is recommended to involve these perspectives as well to gain good understanding of all aspects of family-centred communication in a pandemic. A limitation could be seen in the methodological consideration for interviewing only participants who were supported by FSTs, whereas some participants were also supported by secondary professional support for complicated issues. Referral of relatives with complex issues to secondary professional support, which is often offered by spiritual caregivers or medical social workers, has been normal practice before this pandemic. Hence, little is known about the differences between experiences regarding this secondary care and the support by FSTs.

## Conclusions

Family support in times of the extreme COVID-19 situation and restrictions is very important, as relatives are restricted in communication and have a strong need for information and support. Relatives highly appreciated the FSTs and felt encouraged by structure, frequency, support and understanding by FSTs. However, remote family support should be tailored to the needs of relatives. A fixed contact person on de ICU and video calling might be good extra options for family support, also in future post COVID-19 care, but cannot replace physical visits.

## Data Availability

The dataset generated and analysed during the current study is not publicly available due to privacy of participants and recognizable situations, but are available from the corresponding author on reasonable request.

## Abbreviations

COVID-19: COronaVirusDisease 2019
ICU: Intensive Care Unit
FST: Family support team
PPE: Personal Protective Equipment
UMC: University Medical Center
HTK: Hanna T. Klop
TWK: Tjitske W. Klinge
MN: Mana Nasori
HRWP: H. Roeline W. Pasman
RH: Rianne Hoopman
MAV: Mirjam A. de Vos
BDOP: Bregje D. Onwuteaka-Philipsen
LZ: Lia van Zuylen
CP: Chantal du Perron
MS: Monique Steegers
BT: Birkitt ten Tusscher
FA: Floor Abbink
